# Potassium in the Grape (*Vitis vinifera* L.) Berry: Transport and Function

**DOI:** 10.3389/fpls.2017.01629

**Published:** 2017-09-27

**Authors:** Suzy Y. Rogiers, Zelmari A. Coetzee, Rob R. Walker, Alain Deloire, Stephen D. Tyerman

**Affiliations:** ^1^New South Wales Department of Primary Industries, Wagga Wagga, NSW, Australia; ^2^National Wine and Grape Industry Centre, Charles Sturt University, Wagga Wagga, NSW, Australia; ^3^The Australian Research Council Training Centre for Innovative Wine Production, University of Adelaide, Glen Osmond, SA, Australia; ^4^School of Agricultural and Wine Sciences, Charles Sturt University, Wagga Wagga, NSW, Australia; ^5^Agriculture and Food (CSIRO), Glen Osmond, SA, Australia; ^6^School of Agriculture, Food, and Wine, University of Adelaide, Urrbrae, SA, Australia; ^7^Department of Biology-Ecology, SupAgro, Montpellier, France

**Keywords:** potassium, grapevine, *Vitis vinifera*, berry, fruit, ripening, phloem, xylem

## Abstract

K^+^ is the most abundant cation in the grape berry. Here we focus on the most recent information in the long distance transport and partitioning of K^+^ within the grapevine and postulate on the potential role of K^+^ in berry sugar accumulation, berry water relations, cellular growth, disease resistance, abiotic stress tolerance and mitigating senescence. By integrating information from several different plant systems we have been able to generate new hypotheses on the integral functions of this predominant cation and to improve our understanding of how these functions contribute to grape berry growth and ripening. Valuable contributions to the study of K^+^ in membrane stabilization, turgor maintenance and phloem transport have allowed us to propose a mechanistic model for the role of this cation in grape berry development.

## Introduction

K^+^ is a highly mobile macronutrient that is integral to a number of physiological and biochemical processes within plants. K^+^ has a strong role in regulating the membrane potential of the cell and therefore is critical to the uptake of other ions and sugars. It is essential for plant signaling, osmoregulation, maintaining cation-anion balance, cytoplasmic pH regulation, enzyme activation and protein and starch synthesis (Wang and Wu, 2013; Ahmad and Maathuis, 2014; Shabala and Pottosin, 2014). It is also implicated in programed cell death and senescence (Shabala, 2009, 2017), which in the grape berry is variety dependent and linked to ripening disorders late in ripening (Tilbrook and Tyerman, 2008; Fuentes et al., 2010). At the plant level, this cation is involved in photosynthesis and numerous functions related to plant water relations including growth processes, turgor maintenance and phloem transport (Hsiao and Läuchli, 1986; Liesche, 2016). This macronutrient is important to the development of fruit such as the apple (Nava et al., 2008), tomato (Almeselmani et al., 2009), melon (Lester et al., 2010), peach (Song et al., 2015), pepper (Botella et al., 2017) and strawberry (Song et al., 2017) leading to increased fruit size, soluble solids and color. K^+^ is the most abundant cation within the grape berry at all stages of its development (Hale, 1977; Hrazdina et al., 1984; Rogiers et al., 2006a; Martins et al., 2012). In concert with sugar accumulation, it accumulates rapidly during ripening. From an applied perspective, this cation has a strong influence on juice pH and therefore is also important for berry, juice and wine acidity and color (Mattick et al., 1972; Somers, 1977; Boulton, 1980; Walker and Blackmore, 2012). Moreover, high K^+^ can alter microbiological stability and fermentation processes (Walker et al., 1998). Given the importance of K^+^ to plant functioning as well as grape and wine quality, the mechanisms driving the accumulation of this cation though the vine and into the berry compartments is worthy of further exploration.

The grape berry is a non-climacteric fruit and composed of the seeds surrounded by three tissue layers: the endocarp or the tissue surrounding the seed, the mesocarp, also referred to as the pulp or flesh, and the exocarp or skin (Harris et al., 1968; Pratt, 1971; Hardie et al., 1996). K^+^ is present in all three berry compartments and our knowledge of its accumulation in relation to environmental and cultural factors such as nutrient addition, irrigation, canopy manipulation and rootstock selection has progressed considerably (see reviews by Mpelasoka et al., 2003; Kodur, 2011; Martins et al., 2012). Nonetheless, information on the role of K^+^ in the grapevine and the berry specifically, is unclear. Moreover, the physiological, biochemical and molecular mechanisms responsible for K^+^ accumulation into the berry have only recently received attention. By drawing together this and additional information from other plant systems, it is possible to generate novel hypotheses and compose a useful framework for a process-based model addressing the diverse physiological functions of this cation and its mode of transport into grape berries.

## The Location of K^+^ in the Berry

K^+^ accounts for ca 80% of all the cations within a grape berry (Rogiers et al., 2006a). A grape berry can accumulate over 5 mg of K^+^ by harvest and the accrual into the seeds, mesocarp and exocarp is continuous from fruit set to maturity, but most rapid after the onset of ripening (Hale, 1977; Possner and Kliewer, 1985; Williams and Biscay, 1991; Rogiers et al., 2000, 2006b; Ramos and Romero, 2017). The mesocarp harbors the greatest proportion of K^+^ within the berry. In Grenache Noir, this berry compartment accumulated 60% of the total berry K^+^, with 37% allocated to the exocarp and merely 3% to the seeds (Etchebarne et al., 2009). Correspondingly, in Shiraz, 59% and 32% of the total berry K^+^ were attributed to the mesocarp and exocarp, respectively, with the seeds accounting for 6% (Rogiers et al., 2006a). The brush (the vascular and associated parenchyma tissue proximal to the seeds) and the receptacle were also relatively minor sinks for K^+^ (Rogiers et al., 2006a).

On a fresh weight basis, the concentrations within the Shiraz exocarp were 2-fold greater than the mesocarp prior to veraison, but these differences were less obvious by harvest (Rogiers et al., 2006a). A different study on ripe Shiraz berries found skin concentrations six-fold greater than the flesh (Iland and Coombe, 1988). K^+^ concentrations were also two- to seven- fold higher in Riesling, Cabernet Sauvignon and Chardonnay skins at harvest, relative to the mesocarp, for own-rooted vines as well as those grafted onto Ramsey (Walker et al., 1998; Gong et al., 2010). Knowledge on the concentrations within the exocarp are important for red winemaking since the skin is left in contact with the must for a period of time after crushing to enhance anthocyanin extractability. However, excessive extraction of K^+^ may also occur (Walker et al., 1998) and this is thought to contribute negatively to wine quality as K^+^ binds to tartaric acid increasing wine pH and decreasing the pigmented flavylium form of anthocyanins (Hale, 1977; Boulton, 1980; Walker and Blackmore, 2012). K^+^ also contributes to decreasing free acid levels, altering the tartaric acid: malic acid ratio and forming insoluble K bitartrate crystals during fermentation and within the bottle during the aging process (Conde et al., 2007). Many soils of Australian grape growing regions are particularly high in K (Robinson, 1992; Mpelasoka et al., 2003) and this contributes to high pH juice (Rogiers et al., 2016), necessitating costly tartrate additions in the winery (Walker and Blackmore, 2012), especially in the warmer climates where excessive malate respiration is a concurrent issue.

## The Sub-Cellular Location of K^+^

K^+^ is present in the cytosol most often at 100–200 mM in soluble K^+^ (ion) form (Shabala and Pottosin, 2010; Anschütz et al., 2014). During ripening, the vacuoles accumulate glucose and fructose at up to 1 M (Conde et al., 2007), and this coincides with a doubling in vacuolar K^+^ to 40–50 mM (Keller and Shrestha, 2014). This is considerably less than the hypodermal cells of the berry exocarp harboring vacuoles with K^+^ at up to 0.5–1 M (Storey, 1987). Considering that the hexose concentrations are substantial enough to result in the turgor mediated expansion required for growth, the physiological rationale for this increase in vacuolar K^+^ may be related to its potential role in phloem transport. To prevent the cell from collapsing, cytosolic osmolarity is likely balanced by a combination of K^+^ and other ions as well as organic osmolytes. Stable cytosolic K^+^ concentrations are, however, critical for maintaining the activity of enzymes that require K^+^ as a cofactor, such as pyruvate kinase (Armengaud et al., 2009). Apoplastic solutes are significant in grape berries (Wada et al., 2008) relative to other fruit. But there are cultivar differences in apoplastic K^+^ concentrations of the mesocarp (Keller and Shrestha, 2014), possibly a function of ripening related loss in cell vitality and increased membrane leakiness (Tilbrook and Tyerman, 2008) as indicated by reduced electrical impedance within the apoplast (Caravia et al., 2015). Environmental influences such as light intensity can also modulate apoplasmic K^+^ concentration (Shabala and Wilson, 2001). The complexity of determining apoplastic and vacuolar K^+^ concentrations is aggravated by the presence of gradients in apoplastic and vacuolar K^+^ concentrations across the berry, and this is driven by the diverse metabolic storage functions of cells according to their location (e.g., anthocyanin containing vacuoles in the exocarp).

## Long Distance Transport of K^+^

### K^+^ Uptake and Partitioning

The fruit harbor most of the K^+^ within the grapevine (Pradubsuk and Davenport, 2010; Degaris et al., 2016). K^+^ accumulation into the berry is a function of availability and uptake by roots from the soil (Ruhl, 1989; Wang et al., 2010), the extent of K^+^ redistribution from leaves (Coombe, 1992) and mobilization from storage sinks within the woody structures of the vine (Conradie, 1981; Williams and Biscay, 1991). Alkaline soils (pH > 7.0) are associated with reduced K^+^ availability due to potentially increased Ca^2+^ and Mg^2+^ uptake, to the point that K^+^ deficiency can occur (Hannan, 2011). K^+^ uptake is stimulated by warm spring soil temperatures as a result of increased diffusion rates and root activity (Clarke et al., 2015). A rootstock study indicated that uptake of K^+^ was dependent on root traits such as total root length, total root surface area and percentage of small diameter roots (<0.5 mm) (Kodur et al., 2010). Rootstock differences were also apparent in the proportion of K^+^ that was retained in the root systems or moved upward to the shoots in grafted and ungrafted vines, despite similar uptake and transpiration rates (Kodur et al., 2010, 2011).

Soil solutions contain K^+^ concentrations several orders of magnitude lower than the plant (Ma et al., 2012) and thus considerable energy is invested in its uptake. K^+^ is taken up across the plasma membrane of root cells by membrane transporter and channel proteins that can display high and low affinity uptake kinetics (Chérel et al., 2014). Cuéllar et al. (2010) has demonstrated that a low affinity Shaker K^+^ channel VvK1.1 is mainly expressed in the cortex of grapevine roots and appears to be involved in K^+^ uptake from external media. It is likely that more K^+^ transporters will be characterized in roots of the grapevine, similar to other plants.

It is unclear when and by how much K^+^ is remobilized to the fruit from the reserve pools in the woody structures or from temporary storage in the vegetative shoots and leaves. Reserve mobilization from woody stores was estimated to provide less than 10% of the annual nutrient requirements of the vine (Conradie, 1981). From bud burst to bloom, remobilisation from the perennial components contributed to most of the K^+^ within the growing shoots and inflorescences (Pradubsuk and Davenport, 2010), presumably due to the lack of new fine root growth and slow root uptake in the cool soils (Clarke et al., 2015). Subsequently, uptake from the soil occurred from bloom to harvest, but decelerated after veraison (Pradubsuk and Davenport, 2010). The replenishment of the perennial K^+^ storage pools occurs at various periods across the growing season and may also compete with berry development (Pradubsuk and Davenport, 2010). Post veraison, K^+^ was remobilized from the leaves and shoot tips back to the woody tissues (Pradubsuk and Davenport, 2010) and was also reported to support fruit development in Chenin Blanc (Conradie, 1981) and Cabernet Sauvignon (Williams and Biscay, 1991), but this was not evident in Pinot Noir (Schreiner et al., 2006). To elucidate K^+^ transport mechanisms, ^86^rubidium (^86^Rb^+^) was employed as a substitute for K^+^ because of its similar absorption and distribution characteristics (Läuchli and Epstein, 1970). Upon foliar application, ^86^Rb^+^ was transported mainly to the berries at all stages of development, but with greatest intensity after veraison (Zhenming et al., 2008). The extent of K^+^ relocation from the leaves to the fruit is thus likely dependent on the availability and uptake from the soil, mobilization from leaves and the status of K^+^ reserves in the plant. However, plant water status and rates of phloem flow (Epron et al., 2016) are also important to K^+^ relocation, as are leaf microclimate through its influence on the extent of photosynthesis and phloem loading at the source (Liesche, 2016). Finally, sink activity and demand also drive phloem transport processes (Marcelis, 1996) and thus K^+^ relocation.

### Phloem and Xylem Mobility

**FIGURE 1:**
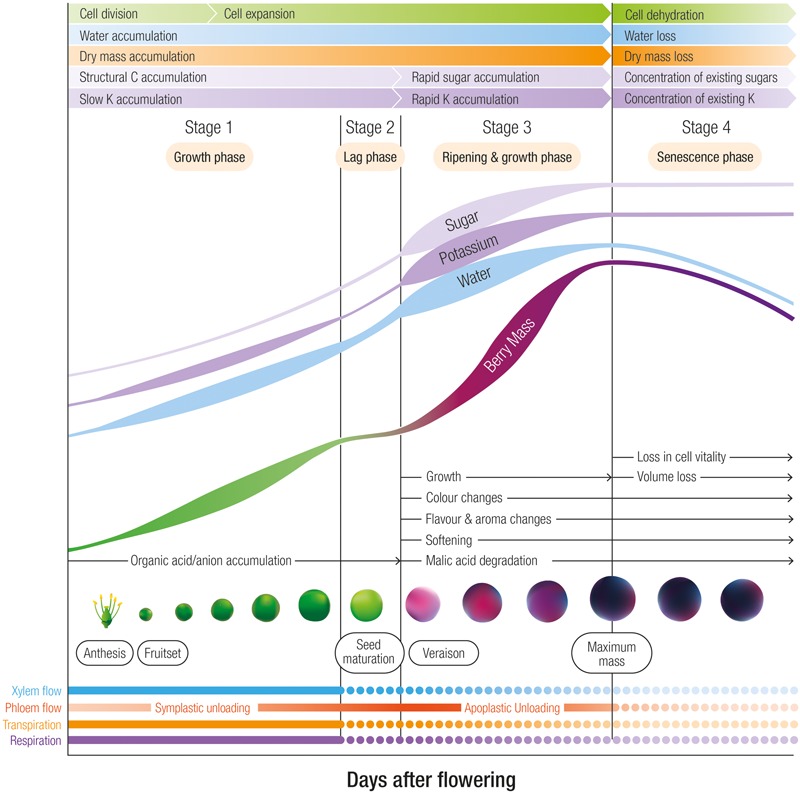
The four developmental stages of grape berries designating phases of rapid sugar, potassium and water accumulation. In Shiraz berries grown in a warm viticulture region of Australia, the lag phase occurs between 45 and 55 days after flowering, and maximum weight occurs at approximately 90 days after flowering. Stage 3 is associated with ripening and includes color, flavor and aroma changes, softening and malic acid degradation. Relative changes in cell division, cell expansion, dry mass, structural carbon accumulation, xylem and phloem flow, transpiration and respiration are also indicated.

K^+^ is classified as an element with high mobility in the phloem (Marschner, 1995). This element is also transported through the xylem but at one 10th the concentrations of the phloem (Keller, 2015). After root uptake and transport through the xylem to the shoots, berry pedicel girdling studies confirmed that the phloem is the predominant route for K^+^ entry into the berry after veraison (Rogiers et al., 2006b). Prior to veraison the daily accumulation rate of K^+^ into Shiraz berries is relatively slow at approximately 20–40 μg per day and then increases 2–4 fold until berries attain maximum weight at which point K^+^ accumulation slows and eventually ceases (Rogiers et al., 2000, 2006a,b) (**Figure 1**). Likewise, in Pinot Noir (Creasy et al., 1993) and Cabernet Sauvignon (Ollat and Gaudillère, 1996), K^+^ accumulation into berries accelerated after veraison. This pattern of initial slow K^+^ entry prior to veraison accords with the estimation by Greenspan et al. (1996) that phloem water contributes to less than 10% of total inflow prior to veraison. With the onset of ripening, however, the authors estimated a 10-fold increase in the rate of phloem flow.

Drivers for the increased phloem contribution and K^+^ accumulation upon ripening include (1) the switch from symplastic to apoplastic phloem unloading (discussed below) and (2) the decline in xylem flow. At veraison, the contribution of the xylem to berry growth appears to decline (Düring et al., 1987; Findlay et al., 1987). The precise reasons for the decline in xylem flow after veraison remain unclear but may be related to a weakening in a hydrostatic gradient within the berry apoplast (Bondada et al., 2005; Scharwies and Tyerman, 2016) possibly due to low transpiration (Greer and Rogiers, 2009) as a result of decreasing surface conductance and the occlusion of the stomata with waxes (Blanke and Leyhe, 1987; Mullins et al., 1992; Rogiers et al., 2004). All things considered, variety differences and environmental factors such as irrigation and the daily variation in the evaporative gradient between the berry and the atmosphere may explain the inconsistent xylem flow patterns. Changes in berry transpiration as well as the reduced xylem contribution to the water and nutrient economy of the berry undoubtedly have large consequences on phloem transport and unloading mechanisms and thus K^+^ accumulation into the berry.

### Cessation in K^+^ Accumulation during Late Ripening

As berries attain their maximum weight during the later stages of ripening, K^+^ accumulation slows. The cessation in phloem flow, and thus sugar and K^+^ import into the berry may be related to an upper limit in the rising osmotic pressure within the vacuole (Patrick and Offler, 1996). The termination of phloem flow also coincides with the occurrence of cell death in the mesocarp. A loss of membrane integrity in mesocarp cells was initially proposed to begin at the onset of ripening (Lang and Thorpe, 1989; Lang and Düring, 1991). However, careful assessment of a number of varieties with vitality stains indicates it does not begin until late in ripening, initiating at the brush and the locular region near the seeds (Krasnow et al., 2008; Tilbrook and Tyerman, 2008; Clarke et al., 2010; Fuentes et al., 2010; Fontes et al., 2011). Even though the vascular tissues appear to remain vital (Tilbrook and Tyerman, 2008), the breakdown in membrane integrity of the mesophyll cells may abolish the compartmentalization between the apoplast and symplast, flooding the apoplast with sugars. Thus the pressure gradient that drives phloem flow is abolished and K^+^ accumulation is arrested.

### K^+^ Transporters and Channels

In plants, the uptake of K^+^ across the membrane is facilitated by channels and transporters either of which can be high or low affinity, and sometimes both (for a full description see Gierth and Mäser, 2007; Alemán et al., 2011; Mitra, 2015). In brief, the channels consist of three families: Shaker type channels, KCO channels and cyclic nucleotide-gated channels (Véry and Sentenac, 2002). These voltage-gated channels are involved in K^+^ uptake from the soil, long distance K^+^ transport in the xylem and phloem and K^+^ fluxes in guard cells. They may be inwardly rectifying (allowing an inward flux of K^+^ into the cell) or outwardly rectifying (allowing a K^+^ outward efflux) based on their voltage dependence of open probability. There are also non-selective cation channels, the molecular identity of which has yet to be determined, though there are many candidates including some aquaporins (Byrt et al., 2016). The transporter category includes TrK/HKT (Na^+^/K^+^ symporter) transporters (Schachtman, 2000), KUP/HAK/KT (H^+^/K^+^ symporter) transporters (Kim et al., 1998), an K^+^/H^+^ antiporter homolog (Bassil and Blumwald, 2014), and glutamate receptors (Véry and Sentenac, 2002). Most studies have been on *Arabidopsis* or rice, and roots have received considerable attention given their importance in K^+^ uptake (Chérel et al., 2014; Chen et al., 2015). Studies are, however, emerging on economically important fruit species with an emphasis on the reproductive tissues. KUP gene expression was assessed during peach development and two of these, PpeKUP1 and PpeKUP2, mediated K^+^ uptake during the rapid fruit expansion phases of this climacteric fruit (Song et al., 2015). A recent study on the non-climacteric strawberry, also from the Rosaceae, reported the cloning of a K^+^ channel gene, *FaKAT_1_* with homology to the *Arabidopsis KAT_1_* and inducible by ABA (Song et al., 2017). Transcripts were expressed in stems, leaves and fruit, increased with ripening and were coupled to the formation of the red color in the flesh.

Like other fruit species, the study of K^+^ channels and transporters in grapevines is still in its infancy, however, to date, three Shaker channels and two KUP/HAK/KT transporters have been cloned and characterized (Pratelli et al., 2002; Davies et al., 2006; Hanana et al., 2007; Cuéllar et al., 2010, 2013). A Shaker channel, SIRK, is expressed at low levels prior to veraison in the berry pericarp and then is undetectable after the onset of ripening (Pratelli et al., 2002). The two KUP/HAK/KT transporters are also expressed highly in skins of pre-veraison berries (Davies et al., 2006). The only K^+^ uptake system identified thus far that is upregulated at véraison is a Shaker channel (VvK1.2) expressed in the plasma membrane of mesocarp and phloem tissues (Cuéllar et al., 2013). These channels and transporters are described in the relevant sections below.

**FIGURE 2:**
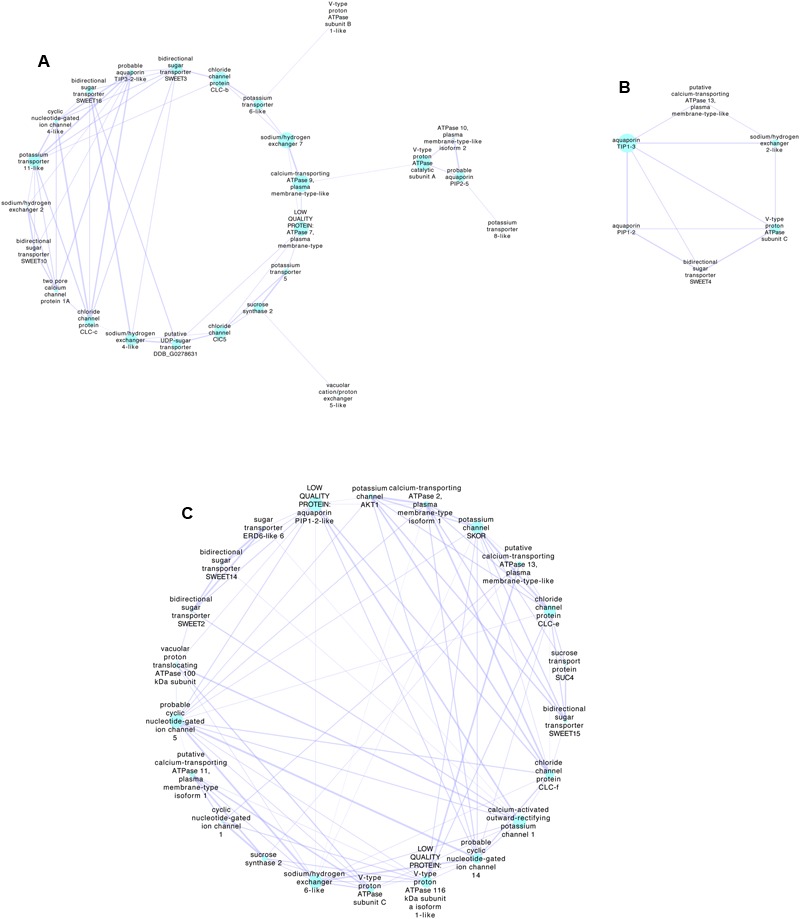
Transporter-gene expression networks for Shiraz berries during development associated with veraison. The networks were obtained using Cytoscape 3.5.1 (http://www.cytoscape.org/index.html) (Shannon et al., 2003) with the RNAseq data of Sweetman et al. (2012), where all transporter genes plus sucrose synthases and invertases were selected giving a total of 260 detected transcripts. Those with very low expression (value of zero during any stage) or those without an associated Genoscope identification were excluded giving a total of 188 transcripts that were log (Ln2) transformed before analysis with Cytoscape (ExpressionCorrelation plugin). Using the classifications of clusters determined by Sweetman et al. (2012), nodes were selected as *Early veraison* or *Veraison up-regulated* or *Veraison onwards*. Networks were then generated from the first nodes connected to these selected clusters using only positive interactions with better than 0.98 (Pearson Correlation Coefficient). Three networks were extracted as shown. **(A,B)** Networks associated predominately with *Veraison upregulated* or *Early veraison*, **(C)** Network associated with *Veraison onwards* that includes the Sweetman et al. (2012) cluster *Increasing*. The size of the nodes indicates the *Betweenness Centrality* of the node indicative of how central the node is in the overall network. The thickness of the edges (lines connecting the nodes) indicates the strength of the positive relationship. Selected highly expressed transcripts related to sugar transport and cation transport, in each network include; **(A)** *bidirectional sugar transporter SWEET10* (SWEET10 VIT_17s0000g00830), *cyclic nucleotide-gated ion channel 4-like* (ATCNGC4 VIT_19s0014g03700), *sodium/hydrogen exchanger 2*(NHX4 VIT_05s0020g01960) and *potassium transporter 11-like* (KUP11 VIT_01s0011g01510). The most highly expressed gene is *probable aquaporin PIP2-5* (PIP2;3 VIT_08s0040g01890). **(B)** *Sodium/hydrogen exchanger 2-like* (VIT_14s0128g00020), *bidirectional sugar transporter SWEET4* (VIT_02s0025g02080). The most highly expressed gene is *aquaporin PIP1-2* (PIP1;3 VIT_02s0025g03390). **(C)** *Bidirectional sugar transporter SWEET15* (SAG29, VIT_01s0146g00260), *probable cyclic nucleotide-gated ion channel 5* (VIT_11s0037g00230), *chloride channel protein CLC-f* (CLC-F VIT_19s0015g01850). The most highly expressed gene is *sucrose synthase 2* (VIT_07s0005g00750). Gene IDs (in brackets) were found by blasting nucleotide sequence from the *Vitis vinifera* 8x assembly (From Genoscope ID provided by Sweetman et al. (2012)) then blasting this nucleotide sequence against the *Vitis vinifera* IGGP_12x in Ensembl Plants. Annotations provided by Sweetman et al. (2012) are in italics.

Co-expression of genes during berry development may provide information on the functional links between proteins. It is possible to examine the gene expression networks from published transcriptomes obtained at different stages of development using tools such as Cytoscape (Shannon et al., 2003). Compiled networks are also available (e.g., Wong et al., 2013). Genes expressed during ripening of Shiraz berries were obtained by Sweetman et al. (2012) using RNAseq. They defined clusters of expression patterns based on expression relative to the key development stage of veraison when rapid sugar and potassium accumulation begins. Similar data sets can also be obtained for other varieties (e.g., Palumbo et al., 2014), but Sweetman et al. (2012) provide more samples at around veraison and have conveniently categorized clusters associated with veraison. Using the Sweetman et al. (2012) data set and extracting all known transporter genes it is possible to then examine co-expression networks on or near veraison. **Figure 2** details three networks extracted using the Cytoscape plugin ExpressionCorrelation. Shown are only the positive interactions with high Pearson Correlation Coefficients (>0.98) based on the premise that for K^+^ and sugar to accumulate the expression of the transporters should be both upregulated at a similar time in development. This does not allow for the possibility of a negative regulator of protein function such as a kinase or phosphatase that were not included in the set of genes use for this analysis, and which are known to regulate transporters. The preliminary analysis shown simply demonstrates the possible genes that could be further examined in detail. Of the set shown some are highly expressed, for example SWEET15 (bidirectional sugar transporter) and various aquaporins. There are several possibilities for K^+^ transport including members of the KUP/HAK/KT family, AKT, cation/H^+^ antiporters and CNGCs. The most highly expressed K^+^ transporter that did not come out of this analysis is AKT2/3, which is upregulated in early veraison (Sweetman et al., 2012) and does have a positive interaction with SWEET10, SWEET2 and SWEET 14.

## K^+^ as an Osmoticum

### Cell Expansion and Growth

Grape berry growth has two distinct growth phases separated by a lag phase (Sadras and McCarthy, 2007). Rapid cell division shortly after fruit set drives cell number and the potential size of the fruit (Coombe and Iland, 2004). However, cell expansion during the majority of stage I and during stage III is the major determinant of berry size and this is mainly due to water accumulation since berries typically contain 75–85% water. Aquaporins are major intrinsic proteins that facilitate the flow of water across membranes and are responsible for most of the hydraulic conductivity of the plasma membrane and tonoplast (Tyerman et al., 2012). Two plasma membrane intrinsic proteins (PIP1 and PIP2) were highly expressed in berries after veraison, corresponding to the period of greatest hydraulic conductivity (Choat et al., 2009). This agrees with earlier results by Picaud et al. (2003) and further functional characterisation will shed light on these processes.

Aquaporins are an important mechanism for controlling water flow through tissues and cells, however, there are still important uncertainties that need to be resolved concerning those key processes that drive water accumulation into berry pericarp cells. It has been suggested that the rapid increase in solute accumulation within the ripening grape berry mesocarp, including sugars, organic acids, K^+^ and other cations such as Mg^2+^, drives osmotic water influx (Mpelasoka et al., 2003). The resulting increase in turgor then extends the cell wall and drives growth (Serpe and Matthews, 2000). While other univalent cations can take over this role, most of them (e.g., Na^+^ and NH_4_^+^) are toxic at high concentrations or not naturally available (e.g., Rb^+^).

A role for K^+^ in driving cell expansion is evident in root hair elongation (Rigas et al., 2001), cotton fiber elongation (Ruan et al., 2001) and *Arabidopsis* shoot expansion (Elumalai et al., 2002; Apse et al., 2003; Barragán et al., 2012). Rootstock studies in grapevines demonstrated a strong positive correlation between vine vigor and berry K^+^ (Walker and Clingeleffer, 2009). In the grape berry, KUP/KT/HAK type VvKUP1 and VvKUP2 K^+^ transporters considered by Davies et al. (2006) showed higher activity during flowering and pre-veraison than post-veraison. Expression was emphasized in the exocarp and so these transporters may be responsible for driving vacuolar expansion in this berry compartment. Continued low expression during the ripening phase indicates that it may contribute to K^+^ homeostasis (Martins et al., 2012). The inward-rectifying SIRK channel identified by Pratelli et al. (2002) was expressed in berry guard cells and possibly the xylem at low levels prior to veraison and it was suggested it may be responsible for guard cell regulation and berry water relations at this early stage. In contrast, a vacuolar cation/H^+^ antiporter encoded by the gene VvNHX1, characterized by Hanana et al. (2007), was found to be expressed in the mesocarp and exocarp cells at high levels during veraison and after veraison. Vacuolar H^+^ pumps are responsible for driving the accumulation of inorganic ions, organic anions and sugars by establishing an electrochemical proton gradient (Terrier et al., 2001). It was suggested that VvNHX1 has an important role in vacuolar expansion and mediates K^+^ vacuolar accumulation to drive water entry into the berry cells. The authors went on to clarify that the rapid accumulation of reducing sugars also contributes to the process of generating turgor and driving cell expansion (Hanana et al., 2007). We suggest that under low sugar conditions in the pre-veraison berry, K^+^ probably serves as the predominant osmoticum to drive cell expansion (Lang and Thorpe, 1989; Huang and Huang, 2001). After veraison, however, sugar loading into the mesocarp vacuoles is rapid and can be 10- fold greater than K^+^ concentrations. K^+^ may be a minor contributor to fruit osmotic potential even after veraison, despite occurring at a 10-fold lower concentration relative to the sugars. This is in part because every K^+^ ion requires a complementary negative ion to maintain a balanced charge and these also contribute to the osmotic gradient. The higher K^+^ concentration that is evident in grape exocarp cells, as outlined above, may be related to its osmotic role. The peripheral bundles are located just beneath the exocarp and it would be expected that the apoplastic sugar concentrations are high in this region due to phloem unloading. K^+^ may be required at higher concentrations in the skin vacuoles to counterbalance the strong osmotic pressure of the apoplast. Further work examining the relative contributions of the typical solutes within post-veraison grape berry mesocarp and exocarp cells is required to determine the importance of K^+^ in driving cell expansion prior to and after veraison.

### Regulation of Berry Turgor

K^+^ may have other roles in the berry related to developmental changes in fruit turgidity. Changes in fruit softness and texture during ripening may attract birds and aid in seed dispersal. From a physiological perspective, the mesocarp cells of ripe berries tend to be less turgid than their younger counterparts, presumably to prevent berries from splitting under high soil water availability and low evaporative demand (Matthews and Shackel, 2005; Thomas et al., 2006; Clarke et al., 2010). Moreover, immature green berries tend to swell and shrink diurnally in accordance with plant water status (Lang and Thorpe, 1989), while ripe berries tend to be less sensitive to daily swings in plant water status (Greenspan et al., 1994; Matthews and Shackel, 2005). This uncoupling of plant from fruit water potential may be the result of the accumulation of solutes within the apoplast. Using a modified centrifugation technique, Wada et al. (2008) found that apoplast solute potential decreased from -0.2 MPa to -4.0 MPa from early to late in development and a number of solutes within this extract, including K^+^, increased markedly prior to veraison. Accordingly, Caravia et al. (2015) noted altered apoplast impedance in Shiraz berries during the later stages of ripening, decreasing proportionally with the extent of cell death. Even though post-veraison berries accumulated more fructose and glucose, K^+^, as a very mobile solute, may also reduce the apoplastic tension, but this is likely to be variety dependent.

### Backflow

The occurrence of shriveling as found in fully mature Shiraz berries on vines grown in warm climates (Coombe and McCarthy, 2000) has been attributed to reduced xylem and phloem flows into the berry in combination with ongoing transpiration and possibly backflow (Tyerman et al., 2004; Keller et al., 2006). Water flow in the reverse direction from fruit to leaves via xylem backflow has been demonstrated using tracers in kumquat (Mantell et al., 1980) and through water budget calculations in apple (Lang, 1990) and cv. Italia grapes (at 50 mm^3^ day^-1^, Lang and Thorpe, 1989) after pedicel girdling. Backflow was also occasionally apparent in post-veraison water stressed Cabernet Sauvignon berries during the day (Greenspan et al., 1994). Moreover, using fluorescent dyes, Tilbrook and Tyerman (2009) were able to provide compelling evidence by visually demonstrating backflow from Shiraz grapes to pedicels during the shriveling phase. Further evidence for backflow was sought through xylem pressure measurements in Shiraz. Rather than the expected very negative pressures within the berry xylem after veraison in berries with low osmotic potentials, xylem pressures approaching zero were evident and it was suggested that this may be the result of high concentrations of apoplastic solutes brought about by a loss in membrane selectivity (Tyerman et al., 2004; Tilbrook and Tyerman, 2008; Choat et al., 2009; Hocking et al., 2016). Because the sugars are close to equilibrium across the plasma membrane, K^+^ may account for what little turgor there is. Alternatively, K^+^ may be one of those apoplastic solutes that plays a role in the abolition of the pressure gradient. Incidentally, backflow leading to berry shrinkage is not necessarily a negative attribute from an applied perspective. It decreases yield but it can concentrate flavor and aromas which is potentially desirable depending on the intended final wine style. Nonetheless, given the implications of backflow for berry turgidity, yield, composition, and potentially phloem water recirculation, further studies characterizing factors and conditions driving reverse water flow are well deserved.

### Pollen Tube Growth

Because of K^+^’s ability to behave as an osmoticum, this cation has control over pollen hydration and pollen tube growth to the ovary (Fan et al., 2001; Rehman et al., 2004) and therefore the success of pollination and fertilization of the ovule. A Shaker K^+^ channel, mediating inward K^+^ channel activity, expressed in *Arabidopsis* pollen and in the growing pollen tube confirmed the importance of K^+^ to pollen competitive ability (Mouline et al., 2002; Zhao et al., 2013). Grape flowers are self-pollinated (Lavee and Nir, 1986) and the anthers often burst shortly before the dehiscence of the calyptra, allowing deposit of the pollen grains on the receptive stigma (May, 2004). The moist surface of the stigma then encourages pollen hydration and the formation of the pollen tube (Meneghetti et al., 2006). Ca^2+^, auxin and GA based signaling stimulate the process with the aid of energy from starch degradation (Gillaspy et al., 1993; Taylor and Hepler, 1997; Franklin-Tong, 1999). The 1000-fold elongation and very rapid rates of pollen tube extension (10.7 μm/min) (Staudt, 1982) would suggest that an osmoticum is responsible for the turgor-driven process. Moreover, once the ovule is reached, the rupture of the pollen tube tip to release the sperm cells is driven by a rapid increase in pressure (Staudt, 1982). Given the role of K^+^ as an osmoticum in pollen tube growth of *Arabidopsis*, K^+^ may, accordingly, have a similar function in grape pollen. Consequently, aside from environmental factors resulting in poor pollen germination (Ebadi et al., 1995), poor fruit set in grapes may also be the result of inadequate K^+^ nutrition or K^+^ partitioning to the flower pollen. Moreover, salinity reduced pollen tube growth in grapevines (Baby et al., 2016) and this might be the result of reduced K^+^ uptake under high Na^+^ (Botella et al., 1997).

### Berry Stomatal Control

K^+^ in guard cells is important for regulating the aperture of stomata. K^+^ is accumulated into the guard cell and this draws in water from the surrounding cells, opening the stomata (Nguyen et al., 2017). K^+^ channel activity is known to be regulated by ABA in guard cells in a Ca^2+^ dependent (Köhler et al., 2003) or independent (Blatt, 2000) manner. K^+^ is thus critical for regulating CO_2_ supply and plant water loss. Grape berries also harbor stomata but at a much lower frequency than the leaves, and as the fruit grows their frequency declines even further and they evolve into non-functional lenticels occluded by wax (Blanke, 1986; Rogiers et al., 2004). Nonetheless, early in development, stomata appear to be functional (Blanke and Leyhe, 1987; Breia et al., 2013) and it was estimated that fruit photosynthesis supplies 10% of the carbon required for fruit development (Ollat and Gaudillère, 2000). The grapevine Shaker channel VvSIRK (stomatal inward rectifying K^+^ channel) identified by Pratelli et al. (2002) has a promotor that is expressed in active guard cells. SIRK transcript quantities were higher pre-veraison, decreasing drastically by the time of veraison likely due to the conversion of the stomata into the non-functional lenticels (Pratelli et al., 2002).

### The Role of K^+^ in Phloem Transport

The growth and maintenance of plant tissues is dependent on the translocation of newly fixed photoassimilates from sources (the sites of synthesis, e.g., mature leaves) to the sinks (the sites of consumption or storage, e.g., roots and berries). An osmotic potential gradient is generated through the loading of solutes into the sieve tubes at the source and unloading at the sink, resulting in a water gradient and a mass flow up to 1 m h^-1^ (Tyree and Fensom, 1970; Daudet et al., 2002; Thompson and Holbrook, 2003). This is referred to as the Münch theory and applies to most plant systems. A modification to this hypothesis ‘the turgor-regulated translocation hypothesis’ has been proposed for grape berries (Lang, 1983; Lang and Thorpe, 1986; Lang and Düring, 1991) where a loss in cell membrane integrity and thus cellular compartmentation at veraison results in apoplasmic osmotic pressure gradients. However, as evidenced by the uptake of fluorescent sugars, membranes do remain intact through most of ripening (Krasnow et al., 2008; Fontes et al., 2011) and it has been suggested that this hypothesis requires re-examination (Dai et al., 2010). Upon exiting the berry vasculature, the phloem solutes are deposited in the vacuoles of the mesophyll cells after traversing the tonoplast. Phloem unloading switches from a passive symplastic route (through plasmodesmata) in green berries to an apoplastic route (into the surrounding cell wall matrix) at veraison for at least one cultivar (Zhang et al., 2006), permitting ripening berries to accrue a high concentration of solutes without constraining phloem influx (Patrick, 1997). The switch to apoplastic unloading is apparent in other fruits that accumulate sugars such as the tomato (Ruan and Patrick, 1995) and apple (Zhang et al., 2004). Considerable progress has been made in understanding sugar accumulating processes into the grape mesocarp cells (Davies et al., 2012). Sucrose is the chief form in which sugar is transported through the phloem and this is hydrolysed by acid or neutral invertases, or sucrose synthase, to glucose and fructose either at or some point beyond the sieve element/companion cell (se/cc) boundary (Davies and Robinson, 1996). Both sucrose transporters (Ageorges et al., 2000; Manning et al., 2001; Zhang et al., 2008) and hexose transporters (Fillion et al., 1999; Vignault et al., 2005; Conde et al., 2006; Hayes et al., 2007; Jaillon et al., 2007) have been identified in grapevines.

Improving our understanding of phloem transport processes has applied relevance because the sugar-acid balance of fruit is one of the main determinants for wine quality. Cooler regions often struggle with low sugar in berries, however, recent problems with excess sugar concentration in warm regions due to climate warming leads to excessively high alcohol. This combined with losses in acidity due to malate degradation and rapid K^+^ accumulation may result in wines with undesirable organoleptic qualities.

### Phloem Loading

K^+^ transport in the phloem is generally from older tissues to growing organs such as new leaves and developing fruits (Mengel and Kirkby, 1987). Much attention has been given to sucrose loading and membrane energization by proton pumping in the phloem at the point of collection (source) (Lacombe et al., 2000), and this has revealed that K^+^ can stimulate sucrose loading in a number of species (Schobert et al., 1998). Accordingly, K^+^ deficiency can result in leaf sugar accumulation and it has been suggested this is due to, along with reduced starch synthase activity, reduced phloem loading and impaired sucrose export (Vreugdenhil, 1985; Cakmak et al., 1994; Zörb et al., 2014). A role for K^+^ channels was apparent in an *Arabidopsis* knock-out mutant of the AKT2/3 K^+^ channel where sucrose loading and sieve element sucrose concentration was reduced (Deeken et al., 2002). Likewise, the putative sucrose transporter ZmSUT1 mediated sucrose/H^+^ symport in the phloem of *Zea mays*, while the phloem K^+^ channel ZMK2 was able to stabilize the membrane potential during phloem loading processes and KZM1 channel mediated K^+^ uptake (Philippar et al., 2003). Phloem loading processes have received little attention in grapevines and it has not yet been established if the mechanism is passive symplastic, active apoplastic or a combination shifting with development or environmental cues. The simultaneous increase in sucrose and K^+^ unloading from the phloem during ripening would suggest that the loading of these two solutes in the leaf minor veins are closely linked.

In some species, however, it has been observed that K^+^ enters the se/cc when sugars are low and exits the se/cc when sugars are high (Smith and Milburn, 1980a,b; van Bel and Hafke, 2005) and this has led to the compensatory potassium uptake theory (van Bel and Hafke, 2005). The observation that increased canopy shading results in grape berries with lower sugar but greater K^+^ content (Smart et al., 1985; Archer and Strauss, 1989; Rojas-Lara and Morrison, 1989; Dokoozlian and Kliewer, 1996; dos Santos et al., 2007) might be explained by the compensatory theory, triggered under sub-optimal light and photosynthesis.

### Long-Distance Phloem Transport and Retrieval

Because K^+^ is the predominant mineral nutrient within the phloem sap of many species, including grapevines, it, regulates the osmotic potential of the phloem sap and thus phloem flow rates (Marschner, 1995). The mechanisms of phloem and K^+^ transport in grapevine have been poorly investigated despite evidence that this cation and its charge balancing anions contribute to the hydrostatic pressure gradient between the collection and the release phloem (Lang, 1983). K^+^ and its associated anions have been hypothesized to maintain the hydraulic pressure gradient along the phloem pathway in *Arabidopsis* (Deeken et al., 2002) and *Vicia faba* (Ache et al., 2001). The long distance pathway between the source and sink is relatively leaky and the solutes require retrieval and reloading to maintain the chemiosmotic gradient (Minchin and Thorpe, 1987; Hafke et al., 2005). While some of the sucrose is used to nourish the surrounding tissues and sinks, the rest is reloaded in a mechanism similar to loading at the source. Recent work in *Arabidopsis* has resulted in an hypothesis wherein K^+^ circulating within the phloem may serve as a decentralized energy store to overcome local energy limitations (Gajdanowicz et al., 2011). K^+^ is loaded in source tissues and then transported along the phloem stream to areas deficient in K^+^. After passage through a K^+^ channel, K^+^ may assist plasma membrane H^+^-ATPases to re-energize the transmembrane phloem loading process, such as in the retrieval of leaked sucrose.

A role for K^+^ was also suggested in a somewhat different perspective on the mechanics of long distance phloem transport. Because sieve pores are partly covered with proteins, the narrow pores create a pressure drop across the sieve plate (van Bel and Hafke, 2005). To counteract this, sucrose may be released into the sieve tube apoplast at the proximal end of the sieve tube and retrieved at the distal end (van Bel and Hafke, 2005). Sucrose/H^+^ symport induced depolarization of the plasma membrane of the se/cc can be rectified by K^+^ channels in order to maintain the proton gradient for sucrose uptake (van Bel and Hafke, 2005).

An alternative hypothesis for K^+^ accumulation into grape berries is related to K^+^’s role as an osmotic facilitator for phloem transport. Unlike sucrose, K^+^ plus anions have low viscosity at high concentration, and perhaps facilitate the flow of phloem sap which would be too viscous with sucrose alone (Thompson and Zwieniecki, 2005). This hypothesis could explain the consistent and rapid simultaneous unloading of K^+^ and sugars into the grape berry.

### Phloem Unloading

Finally, K^+^ may have a role in phloem unloading of sugar. Prior to veraison, most of the imported sugar is metabolized and phloem unloading occurs via a symplasmic route. However, once ripening begins the sugars are stored in the vacuole and the sugar-rich mesocarp cells become isolated from the se/cc complex so that phloem unloading can proceed despite the high concentration of sugars at the sink. After sucrose is unloaded from the se/cc complexes into the apoplasm at the point of release (sink) (Zhang et al., 2006), invertase in the cell wall cleaves sucrose into glucose and fructose and these are then transported across the plasma membrane and tonoplast with the aid of monosaccharide transporters (Agasse et al., 2009). Some sucrose may be cleaved by sucrose synthase in the cytosol or be taken up directly by disaccharide transporters in the vacuole and cleaved there (Davies et al., 2012). Aquaporins in the plasma membrane and tonoplast are highly expressed in expanding cells and upregulated simultaneously with sugar transporters (**Figure 2**). Therefore sugar and water transport may be linked (Fouquet et al., 2008) but little is known about how these transporters are interconnected with K^+^ transport during ripening. The details of mineral transport across the plasma membrane and the tonoplast into the mesocarp cells are not yet well elucidated.

A link between sugar and K^+^ unloading was established in the sinks of castor bean (Ache et al., 2001). The Shaker K^+^ channel VFK1 was highly expressed in cotyledons, flowers, stem and sink leaves. Fructose induced *VFK1* gene activity and the two-electrode voltage-clamp technique indicated that K^+^ controlled the electrical properties of the sieve tube plasma membranes. The proposed mode of unloading depicts that sucrose is released through a sucrose-H^+^ antiport from the se/cc and an apoplastic invertase converts the disaccharide into glucose and fructose. These are then taken up into the sink cell with a monosaccharide-H^+^ symporter, lowering the apoplastic H^+^ concentration and activating the K^+^ channel. Other K^+^ channels and transporters also load K^+^ into the sink cell (Ache et al., 2001). K^+^ channels are linked to sugar unloading in castor bean (Ache et al., 2001), maize (Bauer et al., 2000) and *Arabidopsis* (Lacombe et al., 2000) and similar lines of evidence have recently been established at the molecular level in grapevines (Cuéllar et al., 2013). An inward K^+^ channel belonging to the Shaker family (VvK1.2 under control of specific VvCIPK–VvCBL pairs) was expressed specifically in the plasma membrane of mesocarp and phloem tissues with a strong induction at veraison and thought to play a major role in K^+^ transport (Cuéllar et al., 2013). It was stimulated by acidification of the external medium, and thus is likely regulated by apoplastic pH controlled by plasma membrane H^+^-ATPases. This channel may allow rapid K^+^ retrieval from the apoplast by perivascular and mesocarp cells, decreasing the apoplastic concentration of this cation and thereby favoring its unloading from the se/cc complex. It was suggested that this unloading would stimulate phloem flux toward the sink and thus the accumulation of sugars into the berry (Cuéllar et al., 2013). Although this is feasible, apoplastic sugar unloading itself should stimulate phloem flux and it is uncertain why K^+^ would also be required in this role. Perhaps a more likely scenario is that the K^+^ channel stabilizes the membrane as a result of sugar-H^+^ transporter activities, in a similar system as the castor bean. The accumulation of sugar and water into the vacuoles of mesocarp cells are undoubtedly linked but the function of K^+^ in this complex process requires detailed assessment.

### Phloem Water Recirculation

The unloading of phloem sap into the berry is expected to result in rapid water accumulation but due to low berry transpiration rates (Rogiers et al., 2004; Greer and Rogiers, 2009), it was proposed that the excess water is recycled to the plant through the xylem so that phloem flow is maintained (Keller et al., 2006, 2015). Even though flow through the xylem is reduced after veraison, it does not become hydraulically isolated and maintains the capacity to conduct water as indicated by dye (Keller et al., 2006), microscopy studies (Choat et al., 2009) and detailed hydraulic analysis (Scharwies and Tyerman, 2016). The reported increase in the osmotic pressure in the apoplast with ripening (Wada et al., 2008; Keller et al., 2015) was attributed to the accumulation of hexoses and organic anions, mainly those of malate and tartrate (Keller and Shrestha, 2014) despite the predominance of tartrate in the salt form during ripening (Iland and Coombe, 1988). K^+^, however, decreased in the apoplast of Concord and only contributed 4.6% in green berries and 2.7% in mature berries to the osmotic pressures of the apoplast (Keller and Shrestha, 2014), weakening the role for K^+^ in the recycling of phloem water.

## K^+^ and Programmed Cell Death

Reactive oxygen species (ROS) signaling networks may participate to the regulatory network of fruit development (Pilati et al., 2014) including programmed cell death (PCD) (Van Breusegem and Dat, 2006). At higher concentrations, ROS result in oxidative damage to proteins, lipids and other cellular components (Gadjev et al., 2008). The chloroplast and the mitochondrion are considered to be significant ROS generators but ROS are also produced in other locations within the cell including the plasma membrane, peroxisome and cell wall (Foyer and Noctor, 2003; Asada, 2006; O’Brien et al., 2012). ABA, an important regulator of grape berry development (Coombe and Hale, 1973; Wheeler et al., 2009), can at low concentrations induce antioxidative defense systems (Jiang and Zhang, 2001; Sakamoto et al., 2008); paradoxically, at high concentrations it can stimulate the production of H_2_O_2_ and increase lipid peroxidation (Jiang and Zhang, 2001).

Oxidative stress occurs in a number of fruits and similarly in the grape berry veraison is accompanied by an oxidative burst and the accumulation of H_2_O_2_ in the skin (Pilati et al., 2007). In order to cope with overproduced ROS, fruit have evolved defense mechanisms through the synthesis of phenolics, carotenoids, tocopherols, ascorbate, glutathione (DellaPenna and Pogson, 2006) and free radical enzyme scavenging systems (Rogiers et al., 1998; Sarry et al., 2004; Terrier et al., 2005; Pilati et al., 2014). Chloroplasts appear to be active during early fruit development in grapes, but once the photosynthetic apparatus degenerates (Palliotti and Cartechini, 2001), ^1^O_2_ is likely to be generated in the resultant photo-oxidative conditions (Krieger-Liszkay, 2005). Consequently, analogous to leaves (Kim et al., 2010), K^+^ may facilitate ROS mitigation in the chloroplasts of the berry skin early in ripening.

Mesocarp cell death occurs during late ripening in some grapevine cultivars (Tilbrook and Tyerman, 2008; Fuentes et al., 2010). It is characterized by a breakdown in cell membrane integrity and hypothesized to be the consequence of a form of PCD that contributes to the senescence of the berry. It may be a phenological stage integral to berry ripening and an adaptation to aid in seed dispersal. From a winemaking perspective, the loss in cell membrane integrity aids in juice extractability and may result in the formation of new secondary metabolites important for flavor and aroma (Tilbrook and Tyerman, 2008). The role of K^+^ in these antioxidant systems at the fruit level has not yet received much attention. However, a decrease in the pool of cytosolic K^+^ as a result of efflux through ROS-activated non-selective cation channels, has been linked to the activation of caspase-like proteases and PCD for plants grown under saline conditions (Shabala, 2009).

Further work is required to determine if K^+^ and ROS operate in an interlinked network regulating oxidative processes during berry development. Aside from PCD in the mesocarp, a large number of genes related to proteolysis and autophagy are also upregulated in the grape berry skin at this stage of ripening (Ghan et al., 2017). Research in this direction may be critical to control the process of ripening and senescence, via regulation of expression and operation of appropriate K^+^ transporters in various berry tissues.

## K^+^, ROS and Plant Stress

K^+^ is able to increase resistance to a number of environmental and biotic stresses because it contributes to the detoxification of ROS (Cakmak, 2005). Plants exposed to stresses such as intense light, drought, heat, cold or pathogens suffer from oxidative damage due to an imbalance between the production of ROS and the detoxification by the antioxidant scavenger system (Mullineaux and Baker, 2010). K^+^ homeostasis is integral to the adaptation of plants to these abiotic and biotic stresses and the PCD that is often associated with them (Anschütz et al., 2014). At the plant level, low K^+^ has been linked to an increase in ROS production, most often through impaired photosynthesis, in *Arabidopsis* (Kim et al., 2010) and a number of crops (Wang et al., 2013). In cotton, K^+^ deficiency resulted in premature leaf senescence as a result of higher H_2_O_2_ and malondialdehyde content (Hu et al., 2016). Genes related to peroxidase metabolism were down-regulated in pear grown under K^+^ deficiency, indicating that under high K^+^, the accumulation of excessive oxygenated compounds was held in check by greater peroxidase activity (Shen et al., 2017). In grapevines, soil application of K^+^ to plants grown under drought stress enhanced antioxidant enzyme activity in the leaves (Haddad and Kamangar, 2016). K^+^ is able to inhibit ROS production under drought stress by maintaining photosynthetic electron transport and reducing NADPH oxidase activity (Cakmak, 2005).

Demidchik (2014) proposed that K^+^ efflux through GORK channels may operate as a ‘metabolic switch’ to promote cell repair during stress such as salinity. Because K^+^ activates numerous enzymes involved in normal cell metabolism (Zörb et al., 2014), its absence may deactivate them. This then allows the diversion of limited energy supplies to urgent defense metabolism (Shabala, 2017). It was suggested that this transient system may be appropriate for confined regions such as the root apex under stressful conditions, however, sustained low K^+^ levels within the cytosol may result in PCD. We could extend this theory then to fruit and perhaps suggest a simple scenario where K^+^ prevents PCD during late ripening by maintaining enzyme activity and thus normal cell metabolism. Could PCD then be triggered in late ripening by the efflux of K^+^ and decreased cytosolic K^+^ levels?

### Berry K^+^ and Drought Resistance

The effects of drought stress on grapevine photosynthesis and water relations are well documented as are the consequences on berry development (Dry et al., 2001). Berry composition is determined by the severity and timing of the water stress with low water constraints often resulting in higher sugar concentrations and improved color through smaller berry size and thus an increase in the skin to flesh ratio (Roby and Matthews, 2004). Moderate to severe water stress, especially during ripening, can decrease berry size and sugar accumulation due to an inhibition of photosynthesis (Williams and Matthews, 1990). The consequences of water stress on berry K^+^ concentrations are also likely a factor of its timing and severity (Etchebarne et al., 2009). In some instances, as demonstrated in irrigation studies, grapevines that do not receive irrigation have lower berry K^+^ concentrations (Freeman and Kliewer, 1983; Hepner and Bravdo, 1985; Klein et al., 2000). K^+^ uptake by plants is often diminished in drought due to reduced mobility in the soil and impaired root uptake (Tazawa et al., 2001; Hu and Schmidhalter, 2005; Osakabe et al., 2013). In tomato, a decrease in root hydraulic conductance and transpiration resulted from the down regulation of aquaporin and K^+^ channel transporter activity (Kanai et al., 2011), possibly as a mechanism to aid in water conservation (Smart et al., 2001).

Contrasting to the tomato system, the K^+^ channel VvK1.1 of grapevines is expressed in root cortical cells and to a lesser extent in seed teguments of young berries and, although not developmentally regulated, this K^+^ channel was markedly upregulated with drought and it was suggested it may play a major role in K^+^ loading into berry tissues with water stress (Cuéllar et al., 2010). Likewise, VvK1.2 expression in the berry flesh, phloem tissues and perivascular cells of the vascular bundles was also enhanced by mild plant water stress. Drought stress induced a two- to threefold increase in gene expression of VvK1.2 at and post veraison, further substantiating that K^+^ transport into berries is affected by drought stress (Cuéllar et al., 2013). Drought stressed plants may need additional K^+^ and K fertilization improves water relations and osmotic adjustment in several plant systems (Shabala et al., 2016).

Similar to other plant systems (e.g., Shabala and Pottosin, 2014; Shabala et al., 2016), we suggest that greater K^+^ concentrations in berries decreases berry sensitivity to drought through ameliorating oxidative stress, controlling long-distance phloem transport and maintaining tissue turgor. When cells of any organism are exposed to hyperosmolality, they most often accumulate organic osmolytes such as sugar alcohols and amino acids. This is because organic osmolytes have compatibility with macromolecular structures and do not modify cellular proteins and protein function (Burg and Ferraris, 2008). In grapevines exposed to drought stress, leaf K^+^ concentrations increased more so than organic solutes potentially because salt based osmotica have lower energetic costs (Patakas et al., 2002). Moreover, salts are used in organisms to respond quickly to osmotic shock (Galinski, 1995). Prior to their partial hydraulic isolation, pre-veraison berries are particularly sensitive to dehydration and K^+^ plus associated anions may play a significant role in maintaining turgor. After veraison, it is likely that the organic osmolytes take on this predominant function because berries accumulate sugars at orders of magnitude greater concentrations than K^+^.

### K^+^ and Frost Tolerance in Flowers

Early spring growth in grapevines, that is to say, emerging shoots and inflorescences, are susceptible to frosts as are berries and leaves during autumn (Fennel, 2004). At the cellular level, frost damage is similar to water stress damage in that it leads to cellular dehydration due to ice formation. High concentrations of solutes can protect against freezing by lowering the freezing point of the apoplastic space and the cell solution (Thomashow, 1999). Flower tissues are particularly sensitive to frost and K^+^ may be an important osmoticum at this early, susceptible stage of development. Indeed, as in other crops (Wang et al., 2013), sensitivity to frost damage declined with the availability of soil K^+^ in grapevines (Eifert and Eifert, 1976). Aside from lowering the freezing point, K^+^ may also decrease frost sensitivity by limiting ROS production, especially as a result of impaired photosynthesis (Cakmak, 2005).

### K^+^ and Biotic Stress Resistance

Grape berries are particularly susceptible to insect and fungal attack (Nicholas et al., 1994). K-deficient plants of the major crop species tend to be more susceptible to infection; the addition of K fertilizer decreases insect infestation and disease incidence (Perrenoud, 1990). The critical roles of K^+^ in primary metabolism ensure optimum berry health and that defense mechanisms are fully functioning. It has been proposed that K^+^ encourages strong cell wall development and stimulates phenol production to prevent further infection (Wang et al., 2013). Moreover, the ability of K^+^ to regulate stomatal closure is an important mechanism for warding off infections. The stomata and lenticels are significant entry points for fungi and bacteria, particularly on the rachis, pedicels, flowers and young berries (Whitelaw-Weckert et al., 2011) and proper closure of the guard cells upon their perception is essential to curtailing internal access (Melotto et al., 2006). K^+^ has been implicated in the rapid response mechanism not only to infection but also herbivory-induced wounding because it is such a mobile element at the plant, tissue and cellular level (Marschner, 1995). Voltage regulated ‘gated’ K^+^ channels are particularly useful in this regard as permeation rates through them are more than threefold faster than pumps and carriers (Tester, 1990).

### Viticultural Considerations

From a viticultural perspective, information is required on the optimal K^+^ concentrations in the berry at various stages of growth and development. Given the ability of K^+^ to translocate from roots to leaves and then back to roots, the grapevine is an extremely responsive and adaptable system, capable of maintaining internal homeostasis and driving nutrient flow to areas of greatest demand. The paradox for the grower is to ensure that enough K^+^ is available to maintain normal growth and berry functioning but simultaneously curtail increases in berry pH so that wine quality is maintained. Considering predicted increases in heat and drought periods within warm viticultural regions, as well as reduced water allocations, sustainable cost-effective management options are required. Mild water stress through irrigation strategies can be considered but is complex due to variety differences, climatic variations and soil variability; extreme care is thus required when considering irrigation as a management tool. Rootstocks such as those of the *Vitis berlandieri* parentage (Wolpert et al., 2005) and others (Walker and Clingeleffer, 2009; Walker and Blackmore, 2012) that are low K^+^ accumulators show promise and require additional research attention. The interaction of K with other elements is complex and the use of Ca and Mg fertilization to reduce K^+^ uptake has come up with inconsistent results (Hannan, 2011). Ca itself can have profound effects on berry development (Hocking et al., 2016). This is likely due to soil and climate interactions but does warrant further experimentation. All these approaches may also induce K^+^ deficiency and thus precise recommendations are required that are based on real-time measurements of vine and also berry K^+^ status. Finally, little attention has been given to the role of the bunch microenvironment on K^+^ accumulation, mainly radiation, temperature, evaporative demand and wind speed. This is despite many studies confirming the role of the microenvironment on other attributes such as sugars (Dai et al., 2011), acidity (Sweetman et al., 2009), color (Matus et al., 2009) and aromas (Azuma et al., 2012).

## Concluding Remarks

**FIGURE 3:**
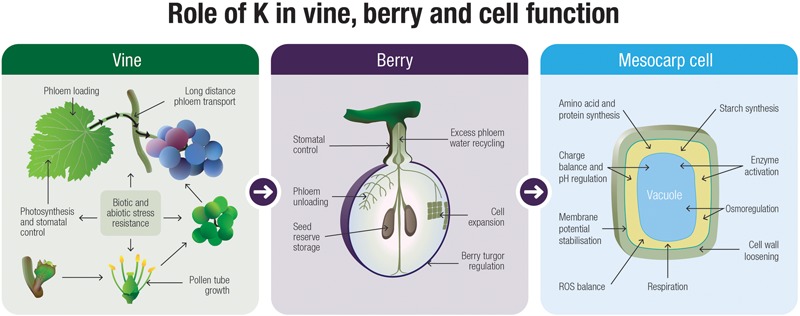
A speculative model describing the functions of K^+^ in the grapevine at the whole-plant, fruit and cellular level.

The basic functions of K^+^ in the developing grape berry have received relatively little attention and there are many uncertainties and gaps in our knowledge. Nonetheless, by examining other plant systems for clues, we are able to build hypotheses on how this cation might influence and even direct fruit development. From **Figure 3** it is evident that K^+^ likely plays a number of roles that are integral to the development of the grape berry and this is one of the main messages that can be derived from this review. There is a large body of literature supporting the role for K^+^ as an osmoticum in driving cell growth and maintaining tissue turgor. Therefore this cation is a likely candidate for driving mesocarp cell expansion in grape berries, especially prior to veraison when sugars are low. The intracellular and long-distance transport of K^+^, mediated by channels and transporters, is critical to fruit ripening but despite little research conducted along these lines in grapevines, some progress has been made. The specific functions for K^+^ in phloem transport is still a matter of debate, however, other crop systems do hint for a role in phloem loading, transport, sucrose retrieval and unloading. In this suggestive backdrop of information, further studies on the molecular determinants of K^+^ transport will undoubtedly provide answers to some of the critical physiological questions relating to long distance K^+^ movement through the plant. Finally, K^+^ has a clear role in biotic and abiotic stress resistance and may co-ordinate responses within the berry through control over ROS and osmotic homeostasis.

The grape berry is an ideal model for understanding the development of non-climacteric fruit due to its wide genetic diversity and ability to thrive in diverse environments. The studies summarized here show that the grape berry is rich in K^+^ and its accumulation is dependent on developmental cues and environmental factors. Further research addressing the biochemical and molecular mechanisms regulating its uptake and partitioning from the sub-cellular to the whole plant scale will help us to gain a broader view of how to manage K in one of the most economically important fruit crops.

## Author Contributions

SR wrote the majority of the paper with input from ZC, RW, AD, and ST. ST provided the data and figures for the Cytoscape analyses. All authors edited and commented on the manuscript.

## Conflict of Interest Statement

The authors declare that the research was conducted in the absence of any commercial or financial relationships that could be construed as a potential conflict of interest.

## Abbreviations

ABA: abscisic acid
ATPase: adenosine triphosphatase
Ca^2+^: calcium ion
H_2_O_2_: hydrogen peroxide
HO^∙^: hydroxyl radical
K^+^: potassium ion
ROS: reactive oxygen species
^1^O_2_: singlet oxygen
O_2_^∙-^: superoxide radical
